# How technostress creators are associated with cognitive overload: the mediating role of emotional exhaustion in academia

**DOI:** 10.1186/s40359-026-05221-x

**Published:** 2026-07-22

**Authors:** Zarqa Farooq Hashmi, Javed Iqbal, Muhammad Zaheer Asghar, Muhammad Naseem Abid

**Affiliations:** 1 School of Education, Huazhong University of Science and Technology, 1037, Luoyu Road, Wuhan, Hubei China; 2 School of Artificial Intelligence for Humanities, Zhejiang International Studies University, Hangzhou, Zhejiang China; 3 Learning and Educational Technology (LET) Research Lab, Faculty of Education and Psychology, University of Oulu, Oulu, Finland; 4 Department of Education, University of Helsinki, Helsinki, 00014 Finland

**Keywords:** Technostress creators, Cognitive overload, Emotional exhaustion, University, Students

## Abstract

**Background:**

Based on the Transactional Model of Stress and Coping and Cognitive Overload Theory, this study explores the association of technostress creators with cognitive overload in academia. Specifically, the authors examined the mediating role of emotional exhaustion on this relationship.

**Method:**

The authors surveyed higher education students from universities in China. We employed a time-lag survey method to test the relationships between the variables used in the research model, utilizing partial least squares structural equation modeling (PLS-SEM) through SmartPLS.

**Results:**

The findings indicate that technostress creators, modeled as a higher-order construct reflected by five lower-order dimensions (techno-complexity, techno-overload, techno-invasion, techno-insecurity, and techno-uncertainty), significantly and positively correlate with cognitive overload and emotional exhaustion. Emotional exhaustion is also significantly and positively associated with cognitive overload. Furthermore, emotional exhaustion was identified as a significant mediator, though this mediating role should be interpreted as data-inferred and requires confirmation through longitudinal or experimental designs. These findings advance the application of the Transactional Model of Stress and Coping and Cognitive Load Theory in digital learning environments. This study offers two key theoretical contributions. First, it extends technostress research by identifying cognitive overload as positively associated with technostress creators among higher education students. Second, it reveals emotional exhaustion as a novel mediating mechanism explaining how technostress creators correlate with cognitive overload. Policymakers should design targeted interventions such as counseling services and stress management workshops to mitigate technostress, while academic institutions and teachers can implement strategies to help students manage technology-related academic stress.

**Conclusions:**

These findings advance the application of the Transactional Model of Stress and Coping and Cognitive Load Theory in digital learning environments. This study offers two key theoretical contributions. First, it extends technostress research by identifying cognitive overload as positively associated with technostress creators among higher education students. Second, it reveals emotional exhaustion as a novel mediating mechanism explaining how technostress creators correlate with cognitive overload. Policymakers should design targeted interventions such as counseling services and stress management workshops to mitigate technostress, while academic institutions and teachers can implement strategies to help students manage technology-related academic stress.

**Supplementary Information:**

The online version contains supplementary material available at https://doi.org/10.1186/s40359-026-05221-x.

## Introduction

Digital technologies are now deeply embedded in higher education through learning management systems, online collaboration platforms, and technology-enhanced learning designs [1, 2]. This can expand access and flexibility, while imposing additional demands on students, including continuous connectivity, platform complexity, frequent technology changes, and the need to manage large volumes of digital information [3, 4]. One challenge that has gained growing attention is technostress, which refers to the stress individuals experience due to their use of technology [5, 6]. Technostress creators, such as techno-overload, techno-complexity, techno-invasion, techno-insecurity, and techno-uncertainty, have been identified as major sources of strain in technology-intensive environments [2, 7]. In academic settings, these stressors may relate to students’ emotional and cognitive functioning by increasing exhaustion and reducing their capacity to process information effectively [8, 9]. Previous studies further show that technology affordances, interactivity, and AI-enabled features can intensify psychological pressure, emotional attachment, problematic use, and cognitive overload among students [10–13]. Recent studies have shown that technostress among students is associated with sleep difficulties, deficient self-control, intolerance of uncertainty, lower academic productivity, reduced academic self-efficacy, resistance to innovation, anxiety, depression, and lower academic motivation [14–20]. Although these studies establish the adverse consequences of technostress, two key issues remain insufficiently explained [21, 22]. First, prior studies have primarily examined outcomes such as academic productivity, burnout, anxiety, and depression, whereas cognitive overload has received far less direct empirical attention. Second, the mediating role of emotional exhaustion in the relationship between technostress creators and cognitive overload remains unclear [21, 23].

Evidence from broader digital contexts further underscores the relevance of examining cognitive strain in technology-intensive learning environments [10–13]. To guide this investigation, we draw on the transactional model of stress and coping and cognitive load theory. The transactional model explains how students may appraise technology-related demands as stressful when those demands exceed their coping resources, which may be associated with emotional exhaustion [24, 25]. Cognitive load theory further suggests that when these demands are associated with the consumption of limited cognitive resources, this may relate to cognitive overload [21, 26]. Together, these theories provide a framework for hypothesizing that emotional exhaustion serves as a mediating mechanism linking technostress creators to cognitive overload. To explain this phenomenon, this study formulates the following research questions:

- Research Question 1: How are technostress creators associated with cognitive overload among higher education students?
- Research Question 2: How does emotional exhaustion mediate the relationship between technostress creators and cognitive overload?

## Literature review

### Research framework

In this study, the proposed model suggests that technostress creators can relate to negative outcomes, such as cognitive overload, through the mediating mechanism of emotional exhaustion. Technostress creators and emotional exhaustion have long been significant concerns among researchers and practitioners due to their role in contributing to cognitive overload [5, 27]. Both technostress and emotional exhaustion are major stressors linked to excessive technology use, potentially leading to cognitive overload [28, 29]. The transactional model of stress and coping contends that cognitive overload results from the interaction between technostress creators and emotional exhaustion among higher education students in a technological environment [24, 25]. Similarly, cognitive overload theory suggests that when learners are exposed to an excessive amount of information beyond their cognitive capacity, it disrupts effective learning [30]. The authors of this study argue that emotional exhaustion mediates the relationship between technostress creators and cognitive overload. The current study empirically examines these relationships by emphasizing the connection between technostress creators and cognitive overload through emotional exhaustion in higher education. In doing so, it extends prior studies that have examined problematic technology use, attachment, interactivity, and emotional strain in digital environments by focusing specifically on the cognitive consequences of technostress in academic settings [10, 11, 31, 32]. This study also builds on previous research by clarifying the role of technostress creators in cognitive overload in the presence of emotional exhaustion.

**Fig. 1:**
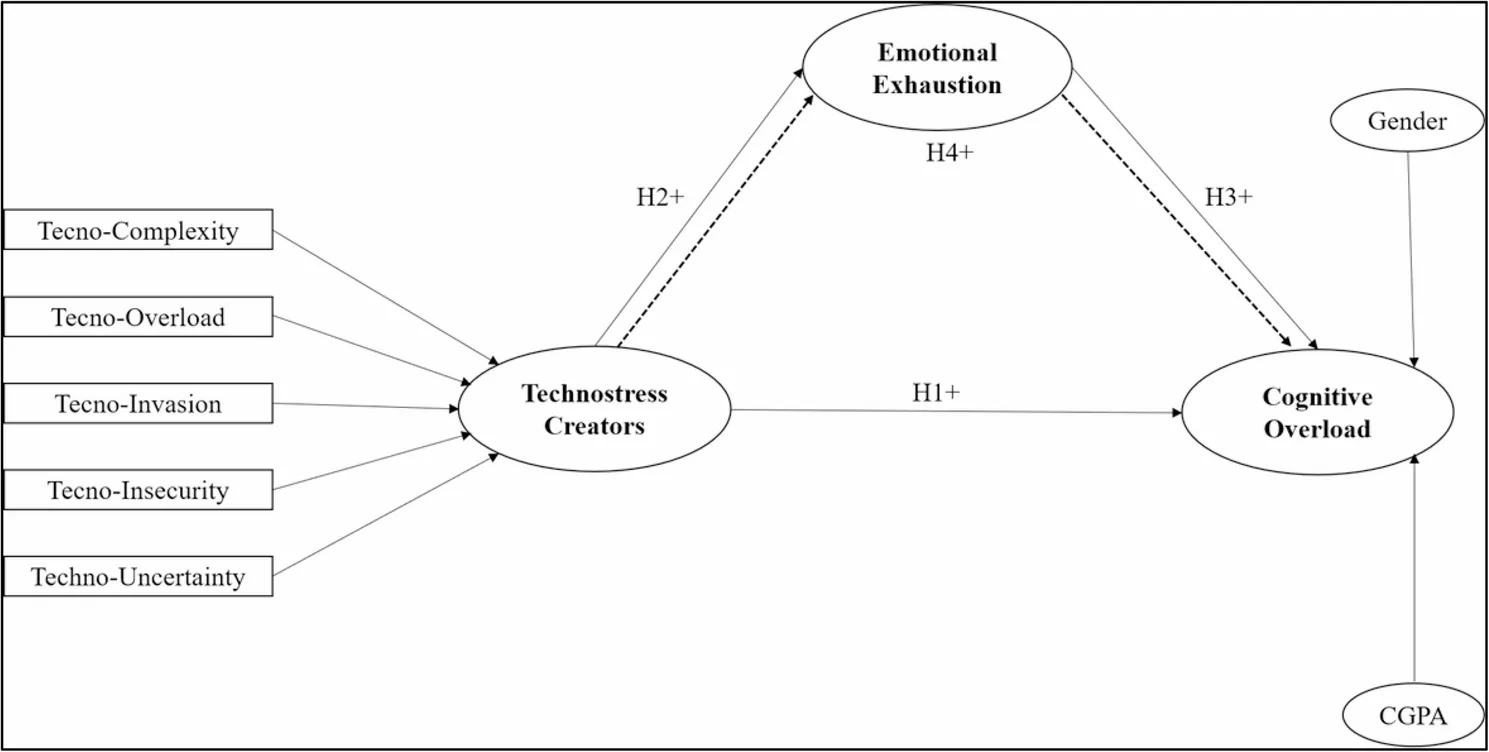
Conceptual framework showing the relationships between technostress creators (techno-complexity, techno-overload, techno-invasion, techno-insecurity, and techno-uncertainty), emotional exhaustion, and cognitive overload, with gender and CGPA as control variables. Note: Solid lines represent direct relationships, while dotted lines represent the mediating relationship

Beyond the technostress literature, studies across various digital platforms suggest that technology-related demands are associated with emotional fatigue, problematic usage, and cognitive strain, supporting the need to examine technostress creators, emotional exhaustion, and cognitive overload in higher education [10, 12, 13, 32]. Existing literature explains that technostress creators significantly correlate with emotional exhaustion among university students [33]. However, universities are also in dire need of developing strategies to mitigate the negative association of technostress on students’ mental health and academic performance [34]. Based on the transactional model of stress and coping, as well as cognitive overload theory, some scholars claim that the technological environment of a university, particularly when it is associated with technostress creators, can have a profound association with students’ mental well-being, which in turn influences their academic performance [21, 26, 30, 35, 36]. In this study, cognitive overload among higher education students may arise from the excessive use of technology, such as the need to process large amounts of information with limited cognitive resources. In line with cognitive load theory and the transactional model of stress, when students face cognitive challenges from processing excessive information, their emotional and personal resources become exhausted, negatively affecting their cognitive abilities and increasing cognitive overload due to technostress creators [21, 26]. The transactional model of stress and coping, together with cognitive load theory, provides the foundation for understanding the association between technostress creators, emotional exhaustion, and cognitive overload [7, 37]. Therefore, our study explores the connections between technostress creators and cognitive overload through emotional exhaustion among higher education students. Figure 1 illustrates these relationships.

### Hypotheses development

#### Technostress creators and cognitive overload

Technostress creators consist of five subdimensions, including techno-overload, techno-complexity, techno-invasion, techno-insecurity, and techno-uncertainty [5]. Rapid technological changes raise serious concerns regarding the adverse association of technostress creators with well-being due to their complexity [38, 39]. Techno-complexity relates to stress, frustration, and mental illness, affecting academic productivity [38]. Techno-overload is an overwhelming information process that may be associated with stress, anxiety, and a sense of being constantly connected to technology, which is associated with impaired mental health [40]. Techno-invasion is the intrusion of academic technology into students’ personal time, creating pressure to stay connected beyond normal study hours and making it difficult to separate study life from personal life [40, 41]. Techno-insecurity deals with sharing personal information online, creating worries and fear among individuals [42]. Techno-uncertainty is characterized by a lack of proficiency in effectively utilizing technology, which is associated with emotional distress [5]. In workplaces, technostress reduces productivity and job satisfaction, while in educational settings, it impedes learning and engagement [43, 44]. The previous studies examined the association of AI-driven interactivity on online user experiences, offering insights into how technological advancements like AI and interactivity can contribute to technostress and cognitive overload, particularly when the user’s flow state is disrupted [28, 29]. Related studies further show that AI functionalities, conversational communication, system interactivity, and perceived user benefits can intensify platform engagement and technology dependence, which may indirectly positively link to cognitive demands on users when digital interaction becomes frequent, immersive, or difficult to regulate [11, 31, 32]. Given that mobile social media and other digital platform use have been linked to psychological burden and cognitive strain [10, 31, 45–47], we consolidated these various platform-related demands into the overarching construct of technostress creators for investigation with cognitive overload. Cognitive overload refers to a condition where the students’ mental resources are exceeded, which is associated with difficulties in processing and retaining information, ultimately impeding their academic performance [27]. Baceviciute, Lucas [48] observed that cognitive overload decreases the capacity to manage information effectively and efficiently, rendering complex information challenging to absorb [49]. Consequently, students face academic challenges, including reduced concentration, heightened ambiguity, ineffective decision-making, weakened problem-solving, and compromised academic performance, endorsed by cognitive overload theory [50–52]. Moreover, cognitive trust in health information sources and the perceived value of that information play crucial roles in fostering healthier information behaviours and reducing anxiety, which positively influences psychological well-being [53, 54]. In the digital space, the quality of mobile social media services and emotional attachment significantly association with user satisfaction and loyalty, while excessive online behaviours, such as social comparisons, contribute to negative psychological outcomes, as highlighted in the study assessing the detrimental influences of fear of missing out on psychological well-being, with self-presentation, upward contrast, and social media stalking serving as moderating factors that contribute to technostress creators [12, 55]. Hence, investigating cognitive overload is imperative for enhancing student well-being and fostering academic success.

Numerous scholarly investigations have demonstrated that technostress creators are associated with cognitive overload, which is associated with reduced creative capacity, critical thinking skills, and learning efficacy attributable to the complex nature of technological software and social media applications [55, 56]. Established technostress creators, including techno-overload, techno-invasion, techno-complexity, techno-insecurity, and techno-uncertainty, have been linked to cognitive strain and reduced academic functioning in technology-mediated environments [57, 58]. Based on the literature using machine learning algorithms, technostress creators are positively associated with cognitive overload, contributing to student learning burnout and affecting perceived academic performance [22]. Furthermore, although prior studies have examined overload, problematic use, inattention, and related digital-behavior outcomes across AI, mobile apps, and social media contexts, the specific relationship between technostress creators and cognitive overload in higher education remains insufficiently examined. Consequently, the current study endeavours to explore the relationship between technostress creators and cognitive overload, formulating the following hypotheses:

- H1: Technostress creators are positively associated with cognitive overload.

### Technostress creators and emotional exhaustion

Emotional exhaustion, characterized by deep-seated fatigue and the depletion of emotional resources, manifests through feelings of tiredness, burnout, pressure, and emotional drainage [59, 60]. This state of exhaustion not only diminishes learning engagement and well-being but also exacerbates cognitive overload [61]. The increasing prevalence of emotional exhaustion and academic burnout from online classes further highlights the detrimental association of technostress in educational settings [62]. To counter these effects, effective coping strategies and simplified system designs are essential. Therefore, understanding emotional exhaustion is crucial for comprehending its connection with cognitive overload as well as technostress creators.

Several studies have emphasized the negative consequences of technostress creators and emotional exhaustion on individuals [1, 63]. Research consistently shows that technostress creators generate stress, anxiety, and emotional exhaustion among learners who rely on technology for their academic tasks [64, 65]. Studies further confirm that technostress creators are closely linked to emotional exhaustion [63, 64]. For example, techno-overload, combined with technical issues such as computer malfunctions and software errors, may be associated with frustration and emotional fatigue as students struggle to complete their assignments within strict time constraints [64]. Weinert, Maier [66] found that social support significantly reduces the effects of technostress creators, which is associated with decreased emotional exhaustion among students. Although prior studies have linked technostress creators to emotional exhaustion, the mediating role of emotional exhaustion in explaining how technostress creators translate into cognitive overload among higher education students remains insufficiently examined. Thus, the current study proposes the following hypothesis to investigate this connection further.

- H2: Technostress creators are positively associated with emotional exhaustion.

### Emotional exhaustion and cognitive overload

Several studies have explored emotional exhaustion and cognitive overload with other variables like well-being and academic performance [67]. Few studies examined the connection between emotional exhaustion and cognitive overload among younger generations in China [68]. Similarly, Wongtongkam [69] and Bayram and Bilgel [70] found that emotional exhaustion is the predictor of stress, sleep difficulty, and depression among students. Based on the above discussion, emotional exhaustion is hypothesized to be positively associated with cognitive overload in higher education settings. Thus, the formulation of the following hypothesis was deemed necessary for investigation within this academic context:

- H3: Emotional exhaustion is positively associated with cognitive overload.

### Mediating role of emotional exhaustion

We hypothesize that the combination of technostress creators and emotional exhaustion among higher education students may also be associated with cognitive overload. Technostress, stemming from the increasing reliance on technology, has become a prevalent issue among higher education students [22]. Concurrently, emotional exhaustion, often arising from academic pressures and technological strain, has been identified as a significant concern [22, 71]. Recent studies indicate that technostress exacerbates cognitive overload, particularly among individuals experiencing high levels of stress [72, 73]. However, the interplay between technostress, emotional exhaustion, and cognitive overload within higher education contexts remains underexplored [22, 74]. This study seeks to address this gap by investigating the mediating effects of emotional exhaustion on the relationship between technostress creators and cognitive overload among higher education students. Thus, we put forward a subsequent hypothesis:

- H4: Emotional exhaustion mediates the relationship between technostress creators and cognitive overload.

### Design and sampling

This study employed a quantitative time-lag survey design to examine the relationships among technostress creators, emotional exhaustion, and cognitive overload. Data were collected in two waves separated by two weeks to introduce temporal separation among the key constructs and reduce the risk of common method bias [75]. The design was used to test the four proposed hypotheses across two data collection points [21, 55]. Responses across the two waves were matched using a participant-specific identifier, and only matched responses were retained for the final analysis.

The target population comprised higher education students enrolled in bachelor’s, master’s, and doctoral programs at six universities in Guangzhou, Guangdong Province, China. Eligible participants were students currently enrolled in higher education programs and actively engaged in technology-supported learning activities. Using convenience sampling, data were collected between March 15 and June 10, 2024, through personal visits that directed participants to an online questionnaire. Convenience sampling was chosen because the study targeted students who were readily accessible through university networks and who had experience using digital technologies for academic purposes. This sampling approach limits generalizability, and selection bias may be present as students who are more active online or interested in technology-related topics may be overrepresented. Additionally, non-response bias was not tested, which weakens confidence in representativeness; this is acknowledged as a limitation of the study.

Participation was voluntary and anonymous. Before completing the questionnaire, participants were informed of the study’s purpose, assured of confidentiality, and asked to provide informed consent. A total of 474 responses were received. Twenty-two responses were excluded due to incompleteness, duplication, or invalidity. After matching responses across the two waves, 452 usable questionnaires were retained for final analysis, resulting in a valid response rate of 95.35%. CGPA was included as a control variable and was self-reported because direct access to institutional academic records was not available. CGPA was included as a control variable because prior research suggests that students with lower academic achievement may experience greater cognitive strain when navigating technology-enhanced learning environments.

### Instrumentation

This study used a structured questionnaire consisting of one demographic section and three measurement sections. Technostress creators were modeled as a second-order construct reflected by five first-order dimensions: techno-complexity (6 items), techno-overload (7 items), techno-invasion (4 items), techno-insecurity (5 items), and techno-uncertainty (4 items). Emotional exhaustion was measured with 5 items, and cognitive overload was measured with 7 items. All substantive items were assessed using a 7-point Likert scale ranging from 1 (strongly disagree) to 7 (strongly agree). The questionnaire items were adapted from previously validated scales and reviewed by six experts to ensure content relevance, clarity, and contextual suitability for higher education students. A pilot test with 60 students was conducted before the main survey to assess item clarity, wording, and contextual relevance, and minor wording changes were made based on participant feedback. The final item wording is presented in Appendix 1.

### Measures

#### Technostress creators

All five dimensions of technostress creators (techno-complexity, techno-overload, techno-invasion, techno-insecurity, and techno-uncertainty) were measured using items adapted from [2]. Techno-complexity (6 items) captures perceived difficulty in understanding and using technology (e.g., “I feel overwhelmed by the complexity of the technology used in my higher education program”). Techno-overload (7 items) reflects the sense of being overwhelmed by excessive information and technology demands (e.g., “I feel like I am drowning in information”). Techno-invasion (4 items) measures technology’s intrusion into personal time (e.g., “I find it difficult to separate study time from personal time because of technology”). Techno-insecurity (5 items) assesses fears of inadequate technical skills (e.g., “I worry that I do not have enough technical skills to keep up”). Techno-uncertainty (4 items) captures confusion about integrating and balancing technology in learning (e.g., “I am unsure about how to integrate technology into my learning practice”). Reliability for all dimensions was acceptable, with Cronbach’s alpha values ranging from 0.774 to 0.848 (see Table 2).

#### Emotional exhaustion

The items used to check the emotional exhaustion level were adapted from the work of Klusmann, Richter [76], and Hunter and Devine [77]. This section comprised five items: “I feel emotionally drained from my studies in higher education, I feel burned out from the demands of my coursework. I feel exhausted even after a good night’s sleep.” Cronbach’s alpha on emotional exhaustion was 0.808. More details related to reliability are presented in Table 2.

#### Cognitive overload

The cognitive overload level measurement scale was adapted from the work of Kim, Park [22] and Iskander [67]. This section comprised seven items: “I often feel overwhelmed by the amount of information”. “I need to process in my higher education.” “I find it difficult to concentrate on my studies in higher education because there is so much to think about.” “I often feel like I have too much to do in my higher education and not enough time to do it all.” Cronbach’s alpha on cognitive overload was 0.822. More details related to reliability are presented in Table 2.

Gender and CGPA were included as control variables because they may influence students’ academic experiences and perceived cognitive strain in digitally mediated learning environments.

### Data analysis procedure

The present study utilized SmartPLS (version 4) and SPSS (version 22) to conduct data analysis. The analysis proceeded in several stages, including descriptive analysis, measurement model assessment, structural model testing, mediation analysis, and multiple-group analysis. In the first stage, descriptive analysis of the demographic data was performed using SPSS. In the second stage, the reliability and validity of the measurement model were assessed through SmartPLS. In the third stage, structural modeling was used to test the research hypotheses. Finally, in the fourth stage, a multiple group analysis (MGA) was conducted to determine whether the effects of technostress creators differed significantly across gender groups. Overall, the time-lag design and selected analytical techniques (PLS-SEM) were appropriate for examining the direct and mediating relationships proposed in the study.

### Common method bias

We implemented two procedural remedies to further mitigate CMV: a 7-point Likert scale was used for survey responses, and a time-lagged design was employed, where data were collected at two intervals. This approach helps to reduce CMV by minimizing respondent bias and separating the measurement of key variables over time. The temporal separation of measurement reduced the likelihood that respondents would use the same immediate cognitive frame when answering all construct items. These steps provided robust measures against potential CMV concerns. In addition to this, we employed Harman’s one-factor test to mitigate standard method variance (CMV) bias [75]. This involved loading all items of the model through exploratory factor analysis (EFA) in SPSS. The results revealed that the first factor of the model explained only 35.224**%** of the total variance, falling below the recommended 50% threshold. Accordingly, the Harman one-factor test did not indicate that a single factor accounted for the majority of the variance, suggesting that common method variance was unlikely to seriously bias the results. In addition to procedural remedies, we conducted a full collinearity assessment (Kock, 2015) as a more robust diagnostic test for common method bias. Following this approach, each latent variable was regressed on all other latent variables in the model. The resulting variance inflation factor (VIF) values were all below the recommended threshold of 3.3, ranging from 1.669 to 2.353. These results indicate that common method bias is unlikely to seriously influence the findings.

### Demographics

Through descriptive analysis, we explain the demographics of our study’s participants in Table 1. Of the 452 respondents, 246 (54.42%) were males, and 206 (45.58%) were females. There were 89 (19.69%) of these higher education students who had an age bracket of 18 to 20 years, 21–23 years, 291 (64.38%), and 24–26 years, 60 (13.27%), and only 12 students (2.65%) had an age larger than 26 years. Moreover, 248 (54.87%) students came from urban areas, and 204 (45.13%) belonged to rural areas. The students who had a CGPA less than 3.00 were 44 (9.73%); a large majority, 366 (80.97%) of these students had a CGPA bracket of 3.10–3.50, followed by those who had a CGPA greater than 3.50, who were 42 (9.29%). Moreover, students enrolled in bachelor’s degree programs were 353 (78.08%), followed by masters were 65 (14.38%) students enrolled in master’s degree programs, and 34 (7.52%) students enrolled in doctoral degree programs. Further demographic details of the participants are presented in Table 1.

**Table 1:** Sociodemographic and academic characteristics of study participants (*N* = 452)

Demographic Characteristic	Number of Participants	Percentage
Gender		
Male	246	54.44%
Female	206	45.56%
Total	452	100%
Age Bracket		
18 to 20 years	89	19.70%
21–23 years	291	64.40%
24–26 years	60	13.30%
Over 26 years	12	2.70%
Total	452	100%
Location		
Urban	248	54.90%
Rural	204	45.10%
Total	452	100%
CGPA Bracket		
Less than 3.00	44	9.70%
3.10–3.50	366	81.00%
Greater than 3.50	42	9.30%
Total	452	100%
Degree level		
Bachelors	353	78.10%
Masters	65	14.40%
Doctoral	34	7.50%
Total	452	100%

### Measurement model

Before testing the structural relationships, we assessed the reliability and validity of the measurement scales. We used Partial Least Squares Structural Equation Modeling (PLS-SEM) with SmartPLS 4 [78]. Although CB-SEM (e.g., AMOS, Mplus) is often used for theory-driven models with large samples, PLS-SEM was selected for two substantive reasons consistent with recent methodological guidelines [79, 80]. First, the primary objective of this study is prediction, maximizing the explained variance in cognitive overload and emotional exhaustion, rather than merely confirming theoretical parameters. PLS-SEM is preferred when prediction is the main goal. Second, our model includes a second-order construct (technostress creators) reflected by five first-order dimensions; PLS-SEM handles hierarchical constructs and complex mediation paths without the convergence issues sometimes encountered in CB-SEM. Thus, the choice of PLS-SEM is appropriate for this study. We first confirmed the reliability and validity of the scales as shown in Table 2.

**Table 2:** Measurement model assessment: reliability and convergent validity of first- and second-order constructs

Scales Used in Model	Indicator Loading	Cronbach’s alpha	Composite reliability (rho_A)	Composite reliability (rho_C)	Average variance extracted (AVE)
Techno-Complexity (TC)		0.896	0.899	0.92	0.659
TC1	0.848				
TC2	0.779				
TC3	0.818				
TC4	0.83				
TC5	0.769				
TC6	0.823				
Techno-Overload (IO)		0.907	0.909	0.926	0.642
IO1	0.813				
IO2	0.786				
IO3	0.775				
IO4	0.805				
IO5	0.801				
IO6	0.821				
IO7	0.810				
Techno-Invasion (TI)		0.839	0.84	0.892	0.675
TI1	0.813				
TI2	0.825				
TI3	0.823				
TI4	0.825				
Techno-Insecurity (TIN)		0.872	0.879	0.907	0.661
TIN1	0.793				
TIN2	0.841				
TIN3	0.816				
TIN4	0.791				
TIN5	0.824				
Techno-Uncertainty (TU)		0.816	0.816	0.879	0.644
TU1	0.812				
TU2	0.806				
TU3	0.798				
TU4	0.794				
Emotional Exhaustion (EE)		0.865	0.866	0.903	0.650
EE1	0.802				
EE2	0.805				
EE3	0.810				
EE4	0.806				
EE5	0.807				
Cognitive Overload (CO)		0.914	0.916	0.931	0.66
CO1	0.832				
CO2	0.826				
CO3	0.798				
CO4	0.829				
CO5	0.808				
CO6	0.788				
CO7	0.803				

Second-order Analysis for Technostress Creators					
Constructs Used in Model	Indicator Loading	Cronbach’s alpha	Composite reliability (rho_a)	Composite reliability (rho_c)	Average variance extracted (AVE)
		0.839	0.87	0.902	0.648
LV scores - IO	0.839				
LV scores - TC	0.833				
LV scores - TI	0.758				
LV scores - TIN	0.835				
LV scores - TU	0.754				

*TC*  Techno-Complexity, *IO *Techno-overload *TI *Techno-Invasion, *TIN*  Techno-Insecurity, *TU*  Techno-Uncertainty, *EE *Emotional Exhaustion, *CO *Cognitive Overload, *rho_A *Dijkstra–Henseler’s rho, *rho_C *composite reliability, *AVE*  average variance extracted, *LV *latent variable

A factor-loading approach was used to measure reliability. This approach explains that the index of each item is above the threshold value of 0.60 [81]. Factor loading is commonly used with various reliability methods such as Cronbach’s alpha, rho_A, and rho_C. For these approaches, the threshold value of 0.70 is generally recommended for assessing reliability [78]. Table 2 indicates that all these approaches are higher than the threshold value and meet the standards required for scale reliability. Hence, it was determined that the scales had met the reliability requirements. We measured convergent validity by applying the average variance extracted (AVE). The threshold value of AVE is 0.50 [81]. The analysis in Table 2 indicates that all scales met the criteria of convergent validity and are considered valid to proceed with the structural analysis (see Table 2).

Henseler, Hubona [82] criticized Farnell’s approach to measuring discrimination validity. They stated that this method is unsuitable for assessing reflective scales’ discrimination validity. They offered an alternate approach, heterotrait–monotrait (HTMT). They suggested a more suitable method [83]. Thus, we used the HTMT to assess discriminant validity by assessing correlations between variables and the items of the same variables [81]. HTMT cut value is less than 0.90. The value above 0.90 is considered weak discriminant validity. Table 3 indicates that each construct on HTMT has a value of less than 0.90, which means the scales met the discriminant validity requirements (see Table 3).

**Table 3:** Discriminant Validity Heterotrait-Monotrait (HTMT)

Model Constructs	Cognitive Overload	Emotional Exhaustion	Technostress Creators
Cognitive Overload			
Emotional Exhaustion	0.565		
Technostress Creators	0.445	0.511	

*HTMT *heterotrait–monotrait ratio

Collinearity problems in SEM analysis were tested using VIF (variance inflation factor) [81]. It indicates no collinearity problems among dimensions, as VIF values were below 5. In PLS-SEM analysis, the model fit analysis is considered suitable for measuring the appropriateness of the model. The leading indicators, such as SRMR (standardized root mean square residual), d_ULS (Divergence from the Unweighted Least Squares), d_G (Divergence Index), Chi-square, and NFI (normed fit index), were used for this purpose [78]. The ideal threshold values for SRMR are below 0.08, for d_ULS, close to 1, for d_G, Smaller values are considered better, for Chi-square < 0.05 (with a large sample, it can be overly sensitive), and for NFI, above 0.90. Table 4 indicates that collinearity was not a concern, as all VIF values were below 5. The model fit indices also suggested a good fit: SRMR = 0.042 (saturated model) and 0.053 (estimated model), d_ULS = 0.337 and 0.539, d_G = 0.113 and 0.141, chi-square = 298.355 and 379.471, and NFI = 0.929 and 0.909, respectively. Since the SRMR values were below 0.08 and the NFI values exceeded 0.90, the model was considered to fit the data well [81].

**Table 4:** Assessment of collinearity and structural model fit

Collinearity Dimensions	VIF-Cognitive Overload	VIF-Emotional Exhaustion	Model Fit		
Cognitive Overload			Model Fit Indices	Saturated Model	Estimated Model
Emotional Exhaustion	1.246		SRMR	0.042	0.053
Technostress Creators	1.251	1.00	d_ULS	0.337	0.539
			d_G	0.113	0.141
			Chi-square	298.355	379.471
			NFI	0.929	0.909

R^2^ has three threshold values: 0.25, 0.50, and 0.75, which are reflected to signify the weak, moderate, and explanatory solid Power of the model. Table 5 demonstrates that both cognitive overload and emotional exhaustion dimensions have moderated explanatory Power.

**Table 5:** Coefficient of determination for the endogenous constructs

Dimensions	*R*²	*R*² adjusted
Cognitive Overload	0.308	0.302
Emotional Exhaustion	0.197	0.195

R² coefficient of determination; adjusted R² adjusted coefficient of determination

The f² values of 0.02, 0.15, and 0.35 indicate small, medium, and large effect sizes, respectively. As shown in Table 6, emotional exhaustion has a medium effect on cognitive overload (f² = 0.170), technostress creators have a small effect on cognitive overload (f² = 0.050), and technostress creators have a medium effect on emotional exhaustion (f² = 0.245) [78].

**Table 6:** Effect size assessment of structural relationships using f²

Variables	Cognitive Overload	Emotional Exhaustion	Technostress Creators
Cognitive Overload			
Emotional Exhaustion	0.17		
Technostress Creators	0.05	0.245	

### Descriptive statistics

Table 7 shows statistical analysis (number, minimum, maximum, mean, standard deviation, kurtosis, and skewness) for the technostress creators, emotional exhaustion, and cognitive overload based on a sample of 452 participants. Table 7 reports the descriptive statistics for the study variables. The mean values were 4.9735 for technostress creators, 4.5726 for emotional exhaustion, and 4.6656 for cognitive overload, with standard deviations of 0.76338, 0.90279, and 0.99911, respectively. The skewness values ranged from − 0.275 to 0.050, while the kurtosis values ranged from − 0.288 to -0.017, indicating that the data were approximately normally distributed.

**Table 7:** Descriptive statistics for the study constructs

Measures	*N*	Minimum	Maximum	M	SD	Kurtosis	Skewness
Technostress Creators	452	1	7	4.9735	0.76338	-0.017	-0.275
Emotional Exhaustion	452	1	7	4.6656	0.99911	-0.288	0.050
Cognitive Overload	452	1	7	4.5726	0.90279	-0.122	-0.179

*N* sample size, *M*  mean, *SD *standard deviation

### Structural model

In this research, we employ Partial Least Squares Structural Equation Modeling (PLS-SEM) to investigate the intricate relationship between technostress creators and their connections with cognitive overload, with a particular focus on understanding the underlying mechanisms leading to emotional exhaustion in academia [83]. Technostress creators were modeled as a second-order construct composed of five first-order dimensions (techno-complexity, techno-overload, techno-invasion, techno-insecurity, and techno-uncertainty), specified as reflective-reflective using the two-stage approach with latent variable scores from the first-order dimensions as indicators. By applying second-order analysis, we aim to capture the shared variance among these dimensions, offering a more comprehensive perspective on the overarching concept of technostress creators. This approach reduces model complexity by consolidating the five dimensions into a single higher-order construct and aligns with our theoretical framework, suggesting a collective contribution to technostress. The subsequent path analysis investigates the direct and indirect effects of Technostress Creators on cognitive overload and emotional exhaustion, providing valuable insights into the holistic understanding of the complex interplay between technostress and its consequences in the academic context. The selection and evaluation of indicators, coupled with a thorough assessment of model fitness and significance, indicate the robustness and validity of our findings [84].

**Fig. 2:**
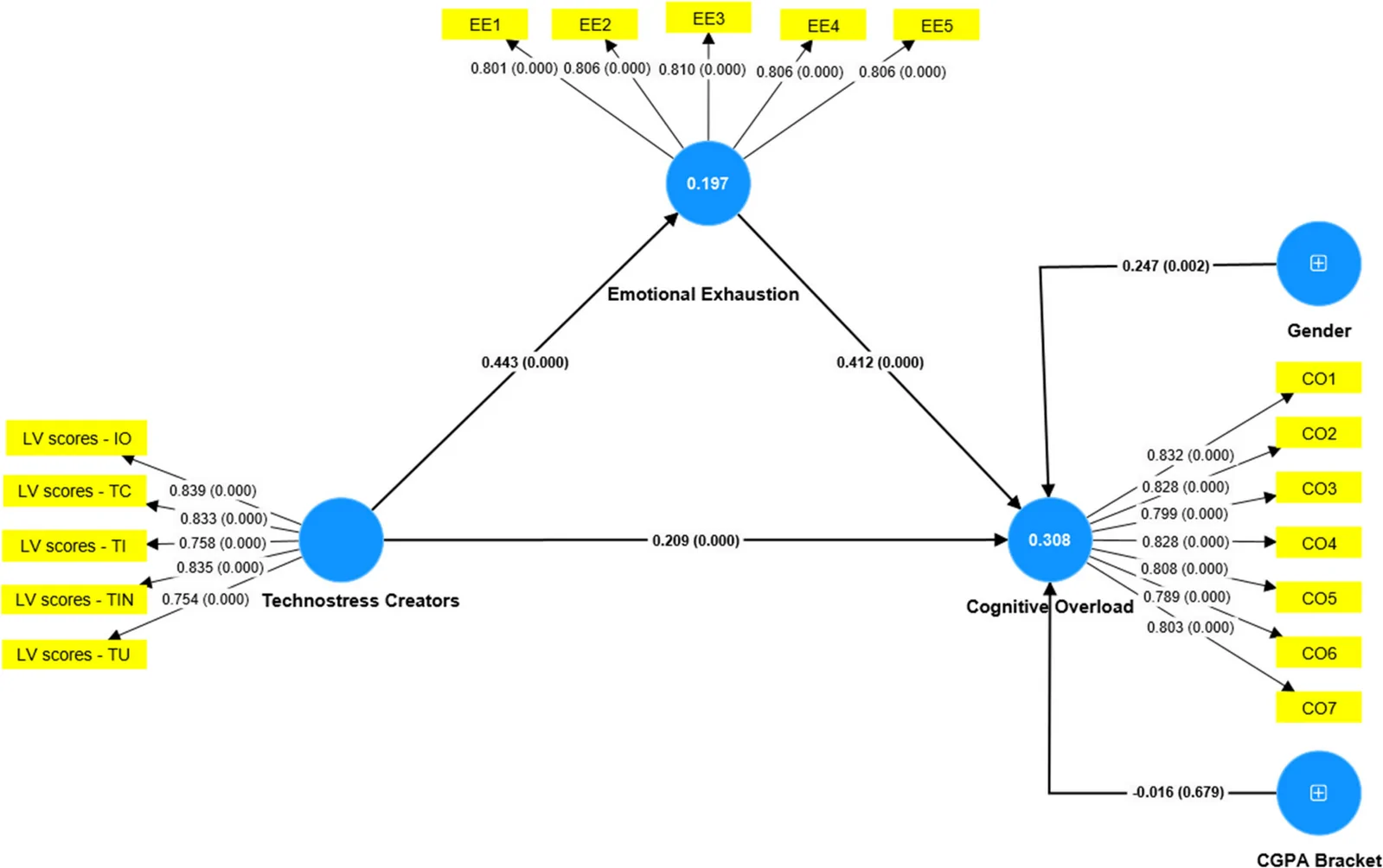
Estimated structural model illustrating the effects of technostress creators (techno-overload, techno-complexity, techno-invasion, techno-insecurity, and techno-uncertainty) on emotional exhaustion and cognitive overload, including standardized path coefficients, indicator loadings, and control variables (gender and CGPA)

Table 8 demonstrates the direct and indirect connections between variables. Technostress creators had a positive, direct, and significant relationship with cognitive overload (*β* = 0.209, *p* < 0.001), which supported H1. This finding indicates that higher levels of technostress creators are associated with greater cognitive overload among students. Similarly, Technostress creators had a positive, direct, and significant connection with emotional exhaustion (*β* = 0.443, *p* < 0.001), which confirmed H2. This suggests that technology-related demands are significantly and positively associated with students’ emotional exhaustion. Likewise, emotional exhaustion had a positive, direct, and significant connection with cognitive overload (*β* = 0.412, *p* < 0.001), which validated H3. This result implies that emotionally exhausted students are more likely to experience difficulty processing academic demands. These findings indicate that technology-related stressors not only directly relate to the students’ cognitive overload but also intensify emotional exhaustion, which in turn is associated with greater cognitive overload. Table 8; Fig. 2 explain these direct connections.

**Table 8:** Hypothesis testing results for direct, indirect, and control-variable effects

H	Hypothesized Direct Effects	β	Mean	SD	t	*P*	Results
H1	Technostress Creators -> Cognitive Overload	0.209	0.209	0.039	5.289	0.000	Supported
H2	Technostress Creators -> Emotional Exhaustion	0.443	0.445	0.04	11.039	0.000	Supported
H3	Emotional Exhaustion -> Cognitive Overload	0.412	0.413	0.041	10.17	0.000	Supported
H	Hypothesized Indirect Effect	*β*	*Mean*	*SD*	*t*	*P*	*Results*
H4	Technostress Creators -> Emotional Exhaustion -> Cognitive Overload	0.183	0.184	0.024	7.591	0.000	Supported
CV	Control Variables Direct Effects	*β*	*Mean*	*SD*	*t*	*P*	*Results*
Control Variables	Gender -> Cognitive Overload	0.247	0.248	0.081	3.033	0.002	Significant
	CGPA Bracket -> Cognitive Overload	-0.016	-0.015	0.038	0.414	0.679	Not Significant

Indirect effects were tested using bootstrapping. Values of *p* reported as 0.000 should be interpreted as *p* < 0.001. Control variables are reported separately from the hypothesized relationships

*β *standardized path coefficient, *Mean *bootstrap sample mean, *SD *standard deviation, *t *t-statistic, *p *p-value

### Mediating effect

The indirect effect of technostress creators on cognitive overload through emotional exhaustion was positive and significant (*β* = 0.183, *t* = 7.591, *p* < 0.001), supporting H4. Because both the direct effect of technostress creators on cognitive overload and the indirect effect through emotional exhaustion were significant, the findings are consistent with partial mediation. This suggests that technostress creators influence cognitive overload both directly and indirectly through emotional exhaustion (see Table 8; Fig. 2).

### Control variables

Two control variables (gender and CGPA) were also part of this research model. We also tested that only gender had a significant and positive effect on cognitive overload (*β* = 0.247, *p* = 0.002), while CGPA had a statistically insignificant effect on cognitive overload (*β* = -0.016, *p* = 0.679) (see Table 8; Fig. 2).

#### Multiple group comparisons on gender

Measurement invariance across gender was assessed using the MICOM procedure [82]. Configural and compositional invariance were established for all constructs, permitting comparison of path coefficients across groups. However, a significant mean difference was observed for cognitive overload (*p* = 0.004), indicating that male and female students differ in their average levels of cognitive overload. This partial invariance does not invalidate the MGA results but is noted as an additional finding.

Multiple group analysis (MGA) was conducted to examine whether the structural relationships differed by gender. The results showed significant gender differences in all three structural paths. The relationship between emotional exhaustion and cognitive overload differed significantly between male and female students (path difference = -0.420, two-tailed *p* = 0.022). Similarly, the effect of technostress creators on cognitive overload differed significantly across gender groups (path difference = 0.502, two-tailed *p* = 0.011). The path from technostress creators to emotional exhaustion also showed a statistically significant gender difference (path difference = 0.213, two-tailed *p* < 0.001). These findings suggest that gender conditions the strength of the proposed relationships in the model (see Table 9).

**Table 9:** MGA Bootstrapping on Gender

Path	Difference (Group_1 - Group_2)	1-tailed *p* value	2-tailed *p* value
Emotional Exhaustion -> Cognitive Overload	-0.420	0.989	0.022
Technostress Creators -> Cognitive Overload	0.502	0.008	0.011
Technostress Creators -> Emotional Exhaustion	0.213	0.000	0.000

### Model robustness

The information pertains to assessing the predictive power of the Partial Least Squares Structural Equation Modeling (PLS-SEM) structural model. The Q²predict values indicate the predictive relevance of each indicator, where values above zero suggest that the model has predictive relevance. The PLS-SEM_MAE (Mean Absolute Error in PLS-SEM) and LM_MAE (Mean Absolute Error in the linear model benchmark) provide measures of predictive accuracy. Overall, the results show varying degrees of predictive relevance and accuracy across the indicators, with several indicators exhibiting positive Q²predict values and small differences between the PLS-SEM_MAE and LM_MAE values, suggesting acceptable predictive performance. In most cases, the PLS-SEM model produced lower prediction errors than the linear model benchmark, indicating satisfactory out-of-sample predictive power. However, a few indicators showed only marginal differences, suggesting that there is still room for further improvement in predictive accuracy. Therefore, these findings provide useful insights for evaluating the model’s predictive performance in PLS-SEM (see Table 10).

**Table 10:** Assessment of Constructs Predicted Power

Constructs Items	Q²predict	PLS-SEM_MAE	LM_MAE	Differences
CO1	0.094	0.957	0.959	-0.002
CO2	0.128	0.929	0.931	-0.002
CO3	0.122	0.883	0.890	-0.007
CO4	0.098	0.962	0.959	0.003
CO5	0.087	0.973	0.972	0.001
CO6	0.068	0.941	0.948	-0.007
CO7	0.077	0.911	0.915	-0.004
EE1	0.135	0.798	0.800	-0.002
EE2	0.110	0.835	0.846	-0.011
EE3	0.132	0.838	0.846	-0.008
EE4	0.131	0.846	0.854	-0.008
EE5	0.110	0.883	0.888	-0.005

The Differences column is calculated as: PLS-SEM_MAE - LM_MAE

## Discussion

This study examined the relationships among technostress creators, emotional exhaustion, and cognitive overload, highlighting significant associations among these variables within the proposed research framework. Most of the studies in this domain have been conducted in advanced nations [85, 86], and the researchers have focused less on exploring these constructs in emerging nations like China. To the best of the authors’ knowledge, this study is among the few to examine how emotional exhaustion mediates the relationship between technostress creators and cognitive overload among higher education students.

The findings indicate that technostress creators have a direct, positive, and significant association with cognitive overload, thereby supporting H1. This finding suggests that higher technology-related demands can be associated with students’ cognitive burden, which is consistent with prior research on technostress and cognitive strain [87]. Similarly, Sharma and Gupta [21] explored that technology-enhanced learning negatively affects feelings, which makes it hard to manage cognitive functions. Likewise, Li and Wang [88] discovered that technostress creators significantly impact teachers’ performance. Moreover, promoting ineffective technology usage and mismanaging technostress can adversely affect cognitive overload among higher education students.

The findings further indicated that technostress creators have a direct, positive, and significant association with emotional exhaustion, thereby supporting H2. This result suggests that persistent technology-related demands correlate with students’ emotional resources, in line with earlier studies linking technostress to exhaustion [1, 16, 56, 64]. Similarly, Stoeckl and Eckhardt [65] found that technostress is an antecedent to emotional exhaustion among students during the COVID-19 pandemic. Likewise, Dahabiyeh, Najjar [89] found that technostress creators influenced teaching exhaustion, affecting productivity. Thus, the constant pressure of using online technologies for learning may be associated with emotional exhaustion among higher education students.

The results also showed that emotional exhaustion has a direct, positive, and significant association with cognitive overload, thus supporting H3. This finding indicates that emotionally exhausted students are more likely to experience difficulty managing academic information and cognitive demands, which is consistent with prior research. For example, Horvat and Tement [61] conducted two studies to examine the connection between cognitive difficulties and functions with emotional exhaustion among students. They suggested that emotional exhaustion may be associated with decreased cognitive function and be associated with cognitive challenges. Likewise, Tzafilkou, Perifanou [90] also found that emotional exhaustion is associated with cognitive overload among students during the COVID-19 pandemic. Hence, it is plausible that emotional exhaustion is associated with disrupted attention focus and cognitive resources, which may relate to cognitive overload among higher education students.

Finally, the results indicate that emotional exhaustion played a significant mediating role in the relationship between technostress creators and cognitive overload, thereby supporting H4. This finding shows that technostress creators are associated with cognitive overload not only directly but also indirectly through emotional exhaustion, which clarifies the mechanism underlying this relationship [88]. Similarly, Khan, Saeed [91] and Sepahvand, Aref Negad [92] investigated the mediated effect of emotional exhaustion on cyberloafing behaviour, creativity, and emotional well-being. Likewise, Tzafilkou, Perifanou [90] explored that engagement in technologically enhanced learning activities, such as online lectures and online communication with teachers and peers, is associated with emotional exhaustion, which indirectly and potentially correlates with cognitive overload. Moreover, this three-way relationship is consistent with the explanation that technostress creators are positively associated with emotional exhaustion, disrupting cognitive functioning, and which is associated with cognitive overload among higher education students.

An awareness campaign is required to highlight that universities can reduce cognitive overload by implementing strategies such as offering targeted digital literacy training [93], optimizing the use of technological tools to minimize techno-overload, providing mental health support to address emotional well-being problems, and encouraging balanced technology use to prevent the negative effects of technostress on students [94]. Additionally, fostering an academic environment that promotes self-regulation and time management can help mitigate the impact of cognitive overload on student learning outcomes [30]. It would be beneficial for universities to analyze technostress creators in order to identify the specific factors contributing to student stress, allowing them to develop targeted interventions that mitigate emotional exhaustion and cognitive overload, thereby enhancing students’ overall academic performance and well-being [95, 96]. Based on these insights, the authors are motivated to focus primarily on understanding the indirect effects of technostress creators on cognitive overload through emotional exhaustion. While the model includes technostress creators as a foundational construct, the hypotheses are specifically designed to explore the mediating role of emotional exhaustion rather than directly hypothesizing about each dimension of technostress creators. This approach allows for a more nuanced understanding of how emotional exhaustion mediates the relationship between technostress creators and cognitive overload, providing deeper insights into the underlying mechanisms at play. However, given the time-lagged design, this finding should be interpreted as consistent with mediation rather than as definitive causal evidence.

### Theoretical contribution

Our study makes three theoretical contributions. First, we extend the Transactional Model of Stress and Coping [24] by identifying emotional exhaustion as a specific mediating mechanism in the technostress-cognitive overload relationship. While prior applications of this model to technostress have primarily treated cognitive appraisal as a direct precursor to outcomes like burnout or performance decrements, our findings demonstrate that technostress creators first deplete emotional resources (emotional exhaustion), which then impairs cognitive processing capacity. This refines the model by positioning emotional exhaustion as a proximal mediator.

Second, we advance Cognitive Load Theory [26, 30] by revealing an affective pathway to cognitive overload. Traditional applications of this theory focus on intrinsic, extraneous, and germane loads arising from task design. Our findings show that emotional exhaustion, a non-instructional factor, significantly amplifies cognitive overload. This expands the theory beyond purely cognitive task features to include emotional states.

Third, by testing these relationships in a Chinese higher education sample, we provide cross-cultural validation of the integrated Transactional Model of Stress and Coping-Cognitive Load Theory framework, extending prior research predominantly conducted in Western or developed Asian contexts.

### Practical implications

The study explored emotional exhaustion as a negative psychological underlying mechanism between technostress creators and cognitive overload. The findings of the study provide useful, practical implications. First, these nuances and understanding of the study help policymakers design specific targeted intervention programs that address students’ challenges, such as providing counseling services, stress management workshops, and well-being initiatives tailored to mitigate the impact of technostress on students’ well-being. Second, the underlying processes highlighted in the study, the management and teachers of academic institutions can develop more effective strategies to support students in managing academic challenges associated with technology use. Given that emotional exhaustion emerged as an outcome of technostress creators and a mediating mechanism that is associated with cognitive overload, academic institutions can formulate guidelines for technology use that balance educational technology with student well-being. These guidelines may involve training programs for educators and students, emphasizing effective technology use and strategies to manage potential stressors. Finally, policymakers in similar contexts may find these insights useful from the study to formulate policies related to technology use that promote a healthy technological environment, considering the potential impact on students’ emotional well-being and cognitive load. This may involve creating guidelines for the design of virtual learning environments, promoting digital literacy, and incorporating well-being considerations into educational technology planning.

### Limitations and future research directions

The research has several limitations. First, the sample was drawn exclusively from undergraduate, master’s, and doctoral students at six universities in Guangzhou, Guangdong Province, China. The Chinese higher education context is characterized by high academic expectations, intensive technology integration, and a strong examination culture, which may be associated with stronger observed relationships. Therefore, the findings may not be generalizable to other educational levels, cultural contexts, or regions with different technological infrastructures and pedagogical approaches. Future research should examine these relationships across diverse populations and educational settings.

Second, we relied exclusively on self-reported measures, which are associated with potential response biases. Future studies should employ a combination of data collection methods, including objective measures of technology use and cognitive performance, and explore additional factors such as social media application usage, technological upgrades, personality characteristics, and virtual relationships that may relate to technostress experiences.

Third, the mediation analysis was conducted using time-lagged data with a two-week interval between waves. While this temporal separation reduces some common method bias concerns, it does not establish true causal sequencing. Therefore, the observed mediating role of emotional exhaustion should be interpreted as consistent with the proposed mechanism rather than as definitive evidence of causation. Future research employing experimental designs or longitudinal studies with longer intervals is needed to investigate the temporal and causal ordering of these relationships.

Fourth, this study did not test whether respondents differed from non-respondents, which weakens confidence in the representativeness of our sample. Future research should conduct non-response bias tests to strengthen generalizability.

Finally, technostress levels may change over time due to advancements in technology, shifts in academic demands, and students’ adaptation to new tools. Longitudinal studies could provide further insights into the evolving nature of technostress and its association with cognitive overload.

## Conclusions

Our synthesized research framework was developed by integrating the Transactional Model of Stress and Coping, Cognitive Overload Theory, and insights from prior literature to test the hypothesized relationships among key variables. This framework guided our exploration of the complex relationships between technostress creators, emotional exhaustion, and cognitive overload in the context of higher education. By combining these theoretical foundations, we aimed to provide a comprehensive understanding of how technostress is associated with emotional exhaustion and cognitive overload among students. The findings revealed that technostress creators are significantly and positively associated with students’ cognitive overload. The results also demonstrated that technostress creators are positively related to emotional exhaustion, which in turn is positively associated with cognitive overload. Importantly, emotional exhaustion was found to play a significant mediating role in this relationship, though this finding should be interpreted cautiously as it is derived from time-lagged, self-report data and does not establish causation. These conclusions underscore the pivotal role of emotional exhaustion in linking technostress to cognitive challenges faced by students, highlighting the need for strategies to mitigate its impact. These findings highlight the need for institutions to consider developing comprehensive strategies to address the challenges associated with technostress and promote students’ overall well-being. By understanding the nuanced interplay between these variables, we can implement targeted interventions, such as counselling services, stress management workshops, and guidelines for effective technology usage, to support students’ academic success and mental health.

## Supplementary Information

Supplementary Material 1.

## Data Availability

The datasets used and/or analyzed during the current study available from the corresponding author on reasonable request.
