# Supplementary material for: How technostress creators are associated with cognitive overload: the mediating role of emotional exhaustion in academia
Source: BMC Psychol. 2026 Jul 22;14:1494. doi: 10.1186/s40359-026-05221-x (PMC13673731; doi:10.1186/s40359-026-05221-x)
Supplement: Supplementary file 1 [file 40359_2026_5221_MOESM1_ESM.docx]

| **Item** | **Recommendation** | **Page & Line** |
| --- | --- | --- |
| 1 | Indicate the study design in the title or abstract | Page 1, lines 20–25 |
| 2 | Explain the scientific background and rationale | Page 2–4, lines 60–140 |
| 3 | State specific objectives or hypotheses | Page 6, lines 190–210 |
| 4 | Present key elements of study design early | Page 15, lines 470–490 |
| 5 | Describe setting, locations, dates | Page 20, lines 630–650 |
| 6 | Describe participants (eligibility, selection) | Page 20–21, lines 650–690 |
| 7 | Clearly define all variables | Page 17–19, lines 520–620 |
| 8 | Data sources / measurement | Page 17–19, lines 520–620 |
| 9 | Bias (efforts to address it) | Page 24, lines 723–739 |
| 10 | Study size explanation | Page 20, lines 640–647 |
| 11 | Statistical methods | Page 23–24, lines 695–722 |
| 12 | Descriptive data | Page 24–25 |
| 13 | Outcome data | Page 34–41 |
| 14 | Main results | Page 38–41 |
| 15 | Other analyses (e.g., mediation, MGA) | Page 40–41 |
| 16 | Key results summary | Page 42–43 |
| 17 | Limitations | Page 47–48 |
| 18 | Interpretation | Page 42–45 |
| 19 | Generalizability | Page 47 |
| 20 | Funding | Page 48 |
